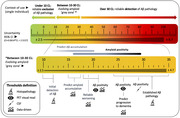# The Centiloid scale: guidance on clinical context of use from the AMYPAD Consortium

**DOI:** 10.1002/alz.086564

**Published:** 2025-01-09

**Authors:** Ariane Bollack, Lyduine E. Collij, Mahnaz Shekari, Santiago Bullich, Núria Roé‐Vellvé, Norman Koglin, Andrew W. Stephens, Mark R Battle, Christopher Buckley, Frederik Barkhof, Juan Domingo Gispert, Gill Farrar, the AMYPAD consortium

**Affiliations:** ^1^ GE HealthCare, Amersham UK; ^2^ UCL Centre for Medical Image Computing, London UK; ^3^ Amsterdam UMC, location VUmc, Amsterdam Netherlands; ^4^ Lund University, Lund Sweden; ^5^ Barcelonaβeta Brain Research Center (BBRC), Pasqual Maragall Foundation, Barcelona Spain; ^6^ Life Molecular Imaging GmbH, Berlin Germany; ^7^ UCL Queen Square Institute of Neurology, University College London, London UK

## Abstract

**Background:**

The Centiloid method (CL) was introduced as a tracer‐independent measure for cortical amyloid load and is now commonly used in Alzheimer’s disease (AD) clinical trials. To facilitate its implementation into clinical settings, the AMYPAD consortium set out to integrate existing literature and recent work from the consortium to provide clinical context‐of‐use recommendations of the Centiloid scale, which has been submitted to the European Medicine Agency for endorsement as a Biomarker Qualification Opinion.

**Method:**

Screening of the literature was performed on the 7/11/23 on PubMed to identify articles mentioning “Centiloid”. The 159 results were reviewed to identify all cut‐offs established based on histopathology, visual reads, fluid biomarkers and cognition. Clinical trials inclusion criteria and amyloid clearance strategies were also included.

**Result:**

Compared to histopathology (gold standard), visual reads, and cerebrospinal fluid (clinical standards), Centiloid quantification accurately reflects the amount of AD pathology. With high certainty at the individual level, a CL value below 10 excludes the presence of Aβ pathology, while a value above 30 is a conservative estimate of amyloid‐positivity (Figure 1). Values falling in between these two cut‐offs (i.e., ‘gray‐zone’) are related to an increased risk of disease progression, but future studies enriched for this population are needed. The AMYPAD consortium additionally assessed the robustness and measurement uncertainty of the Centiloid scale and demonstrated that the method is highly robust against pipeline differences, such as processing space, target region, and reference region, though use of the pons could lead to increased bias and is not recommended. Measurement uncertainty was 3 to 8.3 CL across the total scale and is amyloid burden dependent.

**Conclusion:**

Four clinical context‐of‐use for CL quantification are proposed: 1) adjunct to visual inspection methodology to achieve high certainty regarding the presence or absence of Aβ pathology, 2) a CL level of >30 could be utilised as a conservative cut‐off for the presence of Aβ pathology to guide the management of patients with objective cognitive impairment who may be eligible for anti‐amyloid disease‐modifying therapies, 3) support therapy monitoring and treatment decisions, 4) identification of emerging amyloid deposition and optimize risk‐stratification. Accounting for measurement uncertainty is essential when interpreting CL units.